# Metastatic Cutaneous Angiosarcoma: A Rare Entity

**DOI:** 10.7759/cureus.14577

**Published:** 2021-04-20

**Authors:** Javier Blanco Jimenez, Ghulam Aftab, Dallis Q Ngo

**Affiliations:** 1 Internal Medicine, Saint Peter's University Hospital, New Brunswick, USA; 2 Pulmonary Medicine, Saint Peter's University Hospital, New Brunswick, USA

**Keywords:** cutaneous angiosarcoma, hilar lymphadenopathy, metastatic angiosarcoma

## Abstract

We present a case of a 56-year-old female presented with a chief complaint of four months of dyspnea that had acutely worsened. The patient also reported a chronic right thigh wound.

Chest radiograph on initial presentation demonstrated multiple bilateral rounded opacities. CT pulmonary angiogram demonstrated multiple bilateral rounded nodules seen on chest radiograph along with a right upper lobe pulmonary artery with near-complete compression secondary to large right hilar adenopathy. CT of right lower extremity showed evidence of ulceration with multiple conglomerate subcutaneous masses, predominately anteriorly and laterally.

She underwent a bronchoscopy; a fine-needle aspiration of the subcarinal lymph node was taken. A surgical biopsy from the right thigh wound was also performed. A histological examination from the right thigh ulcer and subcarinal lymph node demonstrated high-grade spindle cell neoplasm, positive for CD-31 and consistent with angiosarcoma.

## Introduction

Angiosarcomas are aggressive, malignant, endothelial-cell tumors of mesenchymal origin [1, 2]. They represent about 2% of all soft tissue sarcomas [3]. Cutaneous angiosarcomas are the most common type. Cutaneous angiosarcoma in the initial stages may resemble benign etiologies and this may lead to a delay in diagnosis. Cutaneous angiosarcoma may metastasize, the most common site of metastasis are the lungs. We present a case of a 56-year-old lady presenting with cutaneous angiosarcoma metastasized to the lung. 

## Case presentation

A 56-year-old female presented with a chief complaint of four months of dyspnea that had acutely worsened in severity over the prior three days. It was associated with subjective fevers and right-sided pleuritic chest pain. Her significant medical history included a 30 pack-year tobacco smoking history, chronic obstructive pulmonary disease (COPD), Ehlers-Danlos syndrome, and obesity class III. Family history was significant for lung cancer in her father who was a heavy smoker. The patient also reported a chronic right thigh wound that began as nodules eight months prior and slowly ulcerated with foul-smelling drainage. As a result of her right thigh wound and associated pain, she had been immobilized for a prolonged length of time. She reported no lower extremity swelling, hemoptysis, or unintentional weight loss.

**Figure 1:**
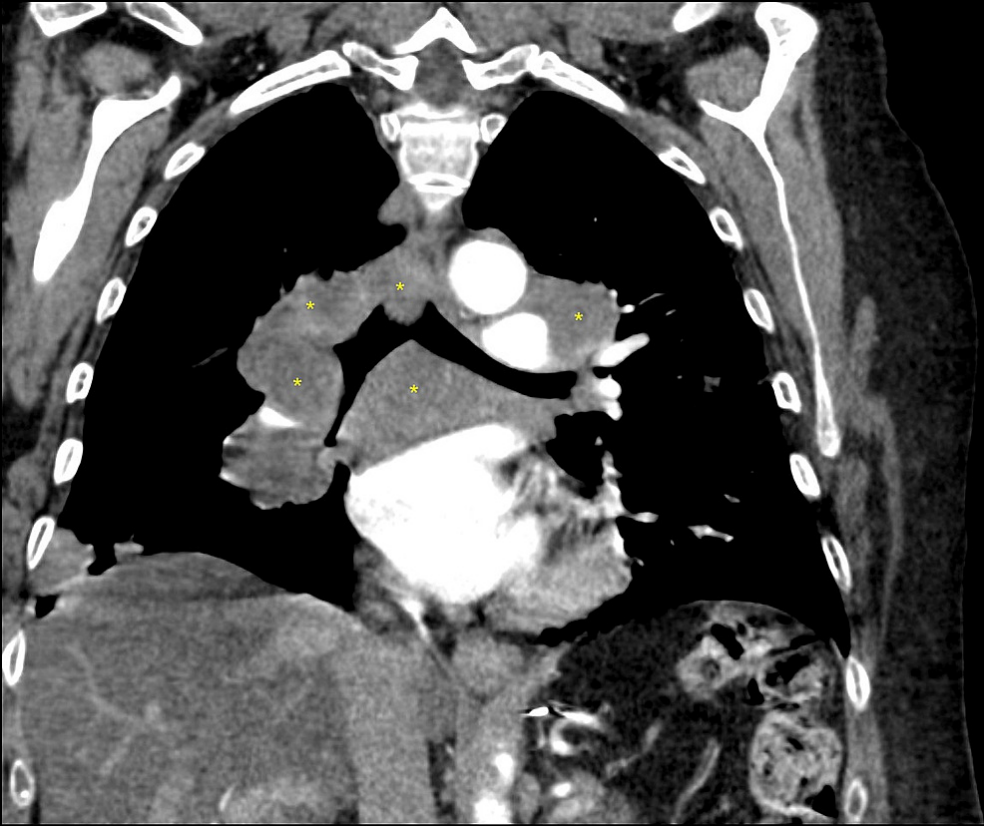
Coronal section of the CT pulmonary angiogram. Bilateral hilar and subcarinal lymphadenopathy causing compression of the right upper lobe, bronchus intermedius, and left upper lobe. Yellow asterisks (*): Enlarged lymph nodes

**Figure 2:**
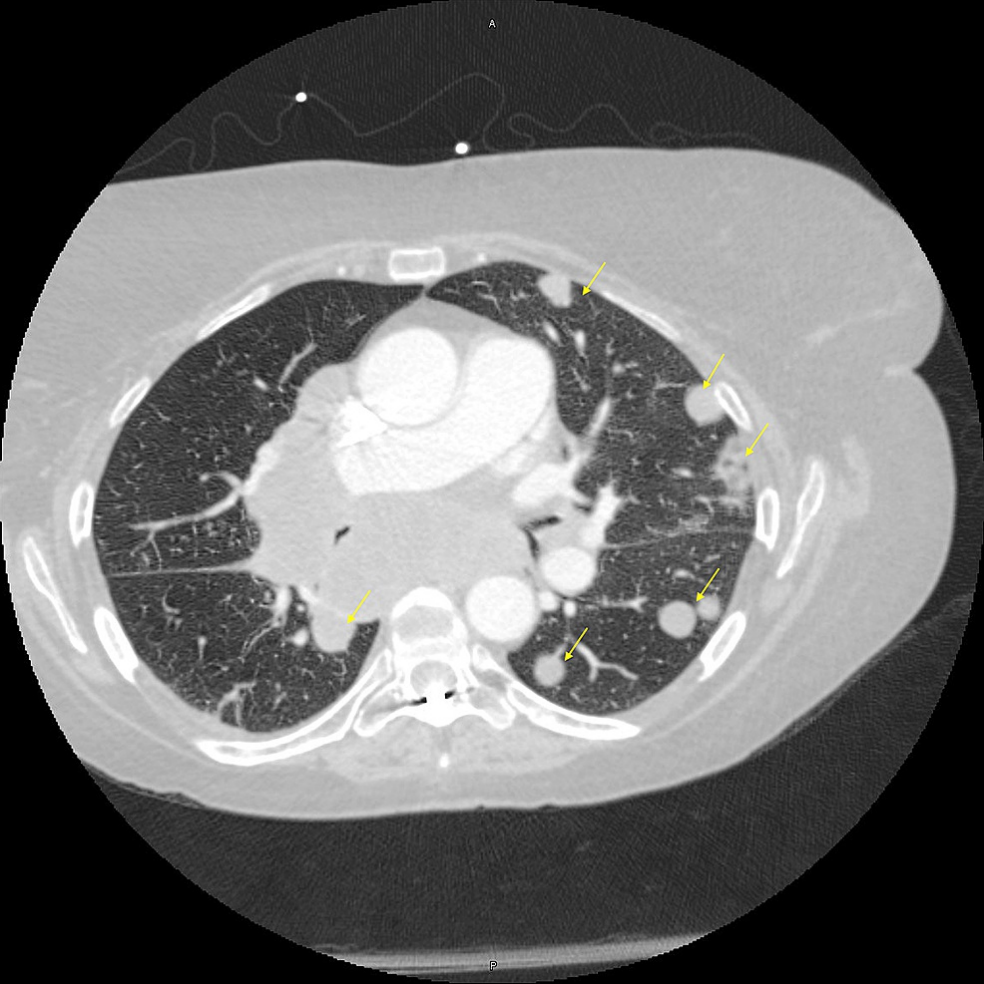
Axial section of the CT pulmonary angiogram. Multiple rounded pulmonary angiosarcoma metastatic lesions. Yellow arrows: Multiple pulmonary nodules

On presentation, she was tachycardic, tachypneic, and hypoxic on ambient air with bilateral expiratory wheezing. Chest radiograph on initial presentation demonstrated multiple bilateral rounded opacities. D-dimer was elevated to 1236 ng/mL D-DU (0.00-211.00), however, CT pulmonary angiogram demonstrated no central pulmonary artery embolus. It did demonstrate multiple bilateral rounded nodules seen on chest radiograph along with a right upper lobe pulmonary artery with near-complete compression secondary to large right hilar adenopathy. There was hilar adenopathy completely encasing bilateral pulmonary arteries, encasement of the left upper lobe segmental pulmonary artery at the hilum with moderate narrowing and mild narrowing of the right middle and right lower lobes. There were multiple enlarged mediastinal, hilar, and subcarinal adenopathies concerning for metastatic disease (Figures 1-2).

**Figure 3:**
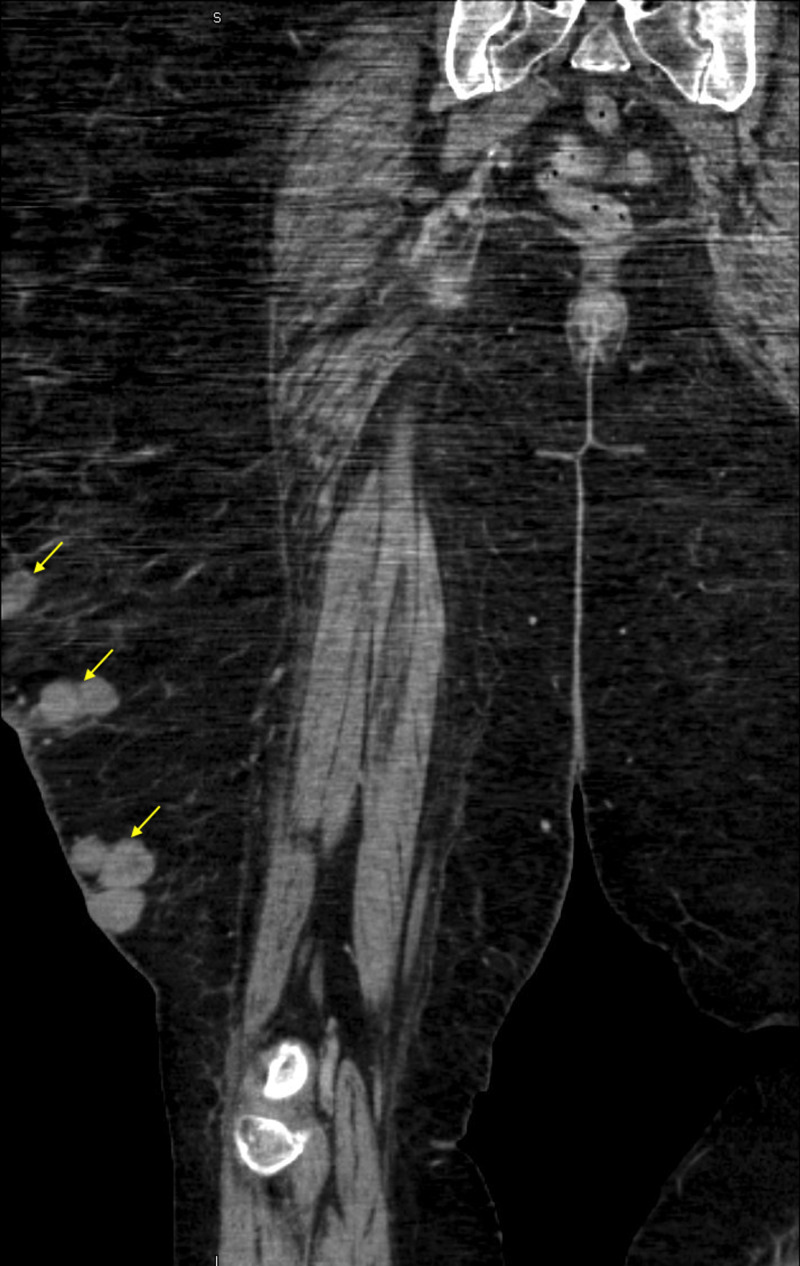
CT Right Lower Extremity with Contrast. Coronal section of the right lower extremity demonstrating multiple conglomerate subcutaneous angiosarcoma lesions. Yellow arrows: Multiple subcutaneous angiosarcomas

**Figure 4:**
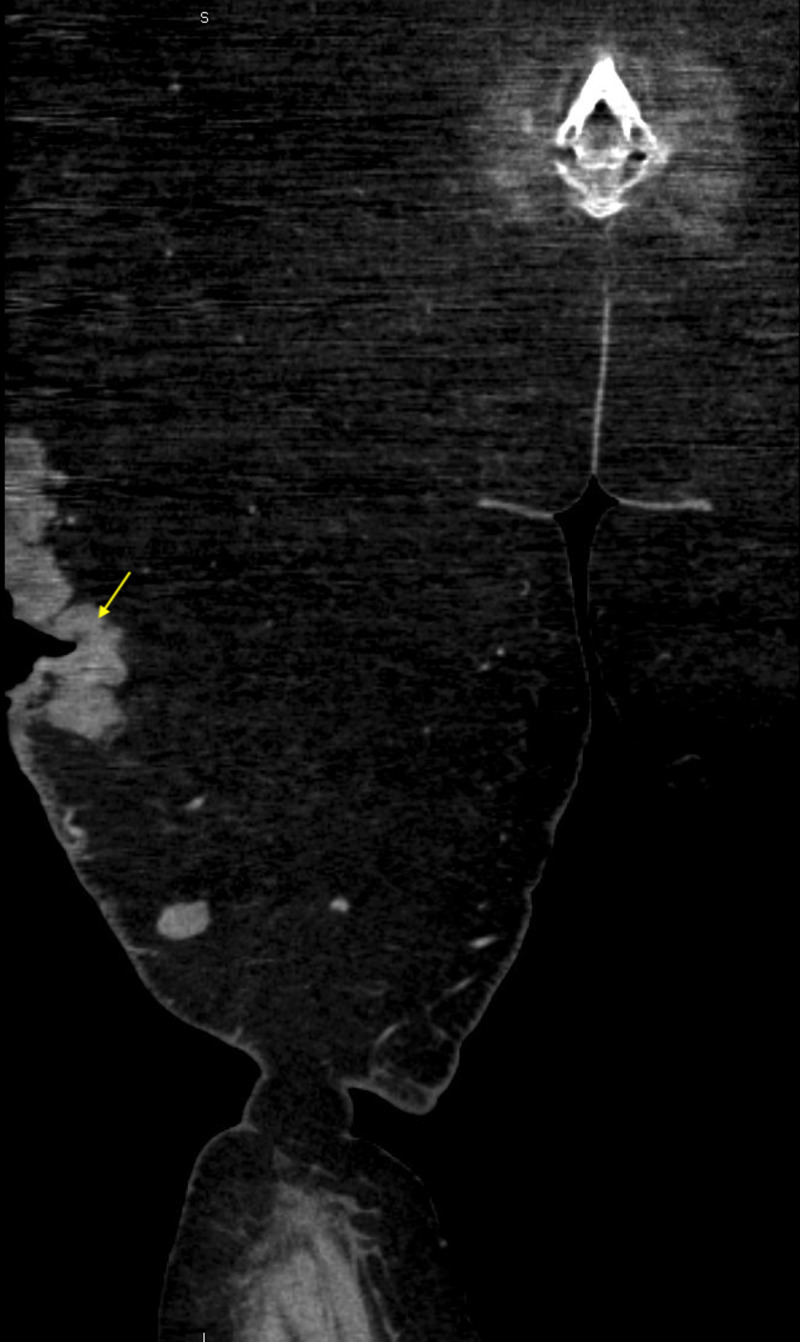
CT scan of the right lower extremity with contrast. Coronal section of the right lower extremity demonstrating subcutaneous angiosarcoma with associated tumor ulceration. Yellow arrow: Ulcerating subcutaneous angiosarcoma

Computed tomography of the right lower extremity showed evidence of ulceration with multiple conglomerate subcutaneous masses, predominately anteriorly and laterally (Figures 3-4).

**Figure 5:**
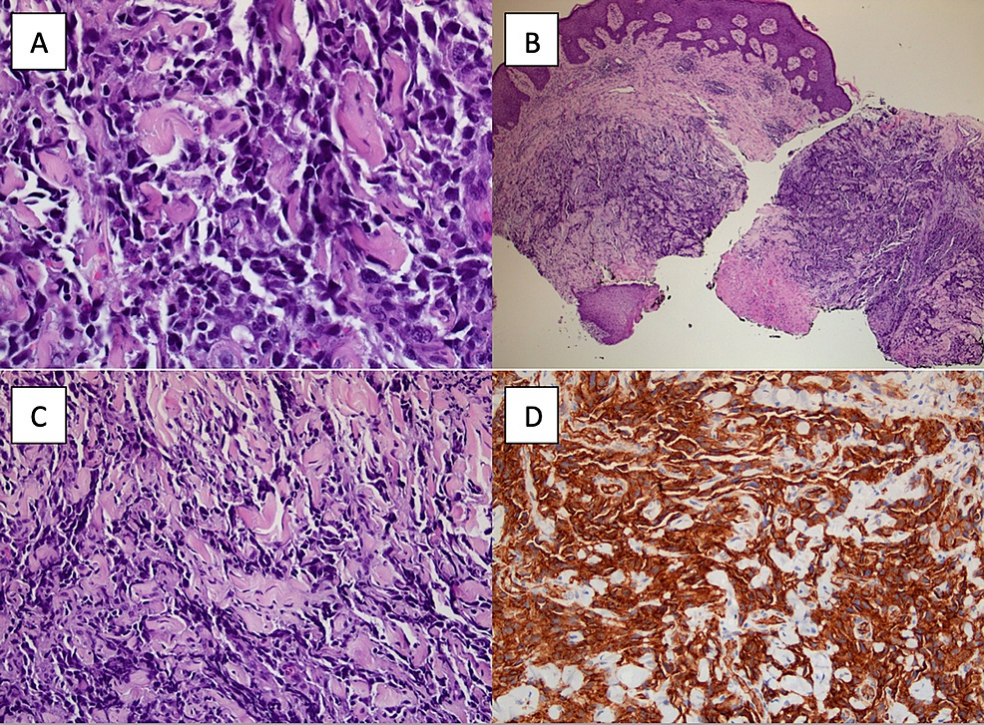
Biopsy showing high-grade spindle cell neoplasm and immunohistochemistry (IHC) positive for CD-31. A. 4x magnification; B. 20x magnification; C. 40x magnification; D. IHC positive for CD-31

The patient developed acute hypoxic respiratory failure non-responsive to non-invasive positive pressure ventilation and required endotracheal intubation. Empiric broad-spectrum antibiotics were started for suspected sepsis. She underwent a bronchoscopy that demonstrated extrinsic compression of the left upper lobe but no lesion was visualized. An endobronchial lesion protruding from the right upper lobe and along the medial wall of the bronchus intermedius was visualized and subsequently dilated. An attempt was made to dilate the right upper lobe, however, endobronchial lesions did not permit dilation. Fine needle aspiration of the subcarinal lymph node was taken. A surgical biopsy from the right thigh wound was also performed. Histological examination from the right thigh ulcer and subcarinal lymph node demonstrated high-grade spindle cell neoplasm, positive for CD-31 and consistent with angiosarcoma (Figure 5).

Despite empiric antibiotics, vasopressors and mechanical ventilation, she continued to decline in health. She received one dose of paclitaxel for angiosarcoma but due to severe multiorgan failure, it had to be stopped and she ultimately expired.

## Discussion

Angiosarcoma is sub-divided into cutaneous angiosarcoma, deep soft tissue, parenchymal tissue (which includes primary-breast angiosarcoma), lymphedema-associated angiosarcoma, and radiation-induced angiosarcoma [4]. Cutaneous angiosarcomas represent 60% of all angiosarcomas and are most common in the head and neck region [5].

Angiosarcoma is more common in individuals between 60 to 70 years of age. Cutaneous lesions are more prevalent in males. The most common risk factors for the development of angiosarcoma are chronic lymphedema and radiation therapy. Familial syndromes have been associated with angiosarcoma. These include hemochromatosis, neurofibromatosis type-1, bilateral retinoblastoma, Maffucci syndrome, and Klippel-Trenaunay syndrome [1]. Angiosarcoma can also be due to chemical toxins. An example is exposure to vinyl chloride, which has been associated with hepatic angiosarcoma [1]. Most metastasis occurs due to the hematogenous spread of tumor cells. Metastases are most common to the lungs, followed by the liver, bones, soft tissue structures, and lymph nodes. Distant metastases develop in more than 30% of patients [6].

Angiosarcoma of the extremities frequently presents as rapidly growing palpable masses [1]. It may also present as violaceous plaques or nodules and resemble rosacea, hemangioma, eczema, and cellulitis [6]. Due to its variable appearance, it may initially be considered a benign lesion. This may lead to a delay in diagnosis. As the tumor enlarges, it can ulcerate and hemorrhage can occur [6].

On CT scan, angiosarcoma presents as an irregular soft tissue mass with contrast enhancement. Angiosarcomas metastasizing to the lungs present as multiple bilateral peripheral nodules on chest radiography. This is a common pattern seen in other cancers metastasizing to the lungs too. CT chest most commonly shows multiple solid nodules. Metastatic angiosarcoma to the lungs may also present as multiple thin-walled cysts. Some people may have a mixed pattern with both nodular and cystic lesions. Rarely, hilar node adenopathy and pleural effusion may be seen. Patients may develop pneumothoraces due to rupture of the cysts [1].

Although most patients present with localized disease, up to 45% have metastatic disease at the time of diagnosis. Angiosarcoma has a 5-year survival of around 35% [4]. Predictors of poor outcome include age over 50, tumor size over 5 cm, male sex, cardiovascular disease, history of smoking, and location in the scalp [6].

For local disease, radical surgery with complete resection is the primary treatment. Wide margins are recommended because of the invasive and multifocal nature of angiosarcomas. Adjuvant radiotherapy with wide treatment fields is recommended as there is a high risk of recurrence [4].

For metastatic disease, chemotherapy is the preferred treatment. The primary drugs used are anthracyclines, ifosfamide, and taxanes. In soft-tissue sarcomas, doxorubicin and ifosfamide used as single agents show a response rate of 16-36%. Combination chemotherapy is associated with increased toxicity but not necessarily with better outcomes. Over the last few years, paclitaxel has shown promising results in angiosarcoma as it has anti-angiogenic activity, with response rates of around 60% [2, 4].

## Conclusions

Chronic ulcers should be investigated as they may represent malignant lesions. Cutaneous angiosarcoma, though rare, should be one of the differentials. This is important as it may help in early diagnosis. This in turn may help improve survival for angiosarcomas. At the same time, it is important for physicians to realize that multiple nodules on CT scan of the chest may represent metastatic lesions.

## References

[1] Gaballah AH, Jensen CT, Palmquist S, Pickhardt PJ, Duran A, Broering G, Elsayes KM (2017). Angiosarcoma: clinical and imaging features from head to toe. Br J Radiol.

[2] Ishida Y, Otsuka A, Kabashima K (2018). Cutaneous angiosarcoma: update on biology and latest treatment. Curr Opin Oncol.

[3] Cooper H, Farsi M, Dorton D, Miller R (2019). Cutaneous angiosarcoma of the leg. Dermatol Online J.

[4] Young RJ, Brown NJ, Reed MW, Hughes D, Woll PJ (2010). Angiosarcoma. Lancet Oncol.

[5] Mendenhall WM, Mendenhall CM, Werning JW, Reith JD, Mendenhall NP (2006). Cutaneous angiosarcoma. Am J Clin Oncol.

[6] Shustef E, Kazlouskaya V, Prieto VG, Ivan D, Aung PP (2017). Cutaneous angiosarcoma: a current update. J Clin Pathol.

